# Intravitreal Bevacizumab in Vitreous Hemorrhage and Diabetes Mellitus

**DOI:** 10.4274/tjo.91328

**Published:** 2017-04-01

**Authors:** Beuy Joob, Viroj Wiwanitkit

**Affiliations:** 1 Sanitation Medical Academic Center, Bangkok, Thailand; 2 Hainan Medical University, China (Visiting Professor)

**Keywords:** Bevacizumab, vitreous hemorrhage, diabetes mellitus

## Dear Editor,

The recent report on “Intravitreal Bevacizumab in Vitreous Hemorrhage and DM” is very interesting.^1^ Alagöz et al.^1^ noted that “intravitreal bevacizumab was found effective in cases with vitreous hemorrhage secondary to proliferative diabetic retinopathy in terms of reducing the need for surgery and increasing the rate of subjects to whom panretinal photocoagulation could be applied in the early period, although there was no impact on final visual acuity”.^1^ There is no doubt that intravitreal bevacizumab can be a good alternative management. However, there are many concerns. First, the cost of intravitreal bevacizumab is high and it is an issue for further assessment of cost effectiveness. Second, although there is no serious complication due to intravitreal bevacizumab administration, subconjunctival hemorrhage is common and becomes an issue for consideration in diabetes mellitus cases.^2,3^ Also, in cases with underlying severe diabetes mellitus, possible unwanted gastrointestinal side effects have been reported.^4^ Onoda et al.^4^ suggested that “ophthalmologists should apply alternative therapies instead of intravitreal bevacizumab to patients with severe diabetes mellitus”.
