# Supplementary material for: Intravitreal Bevacizumab in Vitreous Hemorrhage and Diabetes Mellitus
Source: Turk J Ophthalmol. 2017 Apr 1;47(2):123–4. doi: 10.4274/tjo.91328 (PMC5384120; doi:10.4274/tjo.91328)
Supplement: Related texts

# Reply: RESPONSE FROM THE AUTHORS

**Authors:** Cengiz Alagöz, Yusuf Yıldırım, Murat Kocamaz, Ökkeş Baz, Uğur Çiçek, Burcu Çelik, Halil İbrahim Demirkale, Ahmet Taylan Yazıcı, Muhittin Taşkapılı

**Affiliations:** 1 Beyoğlu Eye Traning and Research Hospital, İstanbul, Turkey

## Dear Editor,

We thank Drs. Beuy Joob and Viroj Wiwanitkit for their commentary on our article “The Efficacy of Intravitreal Bevacizumab in Vitreous Hemorrhage of Diabetic Subjects”. In our study, we found that intravitreal bevacizumab was effective in vitreous hemorrhage secondary to diabetic retinopathy in terms of reducing the need for surgery and increasing the rate of panretinal photocoagulation (PRP) completion in the early period.^1^ It has been demonstrated that treatment with only intravitreal anti-vascular endothelial growth factor (anti-VEGF) in proliferative diabetic retinopathy (PDR) patients did not result in worse results compared to PRP treatment alone in 2 years follow-up; that is, PRP was not found superior to intravitreal anti-VEGF treatment.^2^ Therefore, intravitreal anti-VEGF treatment might be considered as an alternative treatment option for PDR patients, though long-term results are needed. Drs. Beuy Joob and Viroj Wiwanitkit rightly pointed out that intravitreal anti-VEGF treatment is an expensive treatment; however, compared to a vitrectomy surgery, intravitreal bevacizumab is still more cost effective. Like all other intravitreal injection applications, intravitreal bevacizumab may also result in various complications. Subconjunctival hemorrhage is among the most common, but it does not have any impact on visual acuity or the course of the disease. It is well-known that a reduction occurs in serum and plasma-free VEGF levels after intravitreal anti- VEGF injections,^3^ and that there is an increased risk of artherothrombotic events after systemic use of anti-VEGF agents. Fortunately, meta-analysis could not demonstrate any increased risk in clinical practice.^4^ This is mostly because high-risk patients such as subjects with a history of recent myocardial infarction or cerebrovascular event were not involved in the studies. In our clinical practice, we also do not administer any kind of intravitreal anti-VEGF treatment to those high-risk patients.

**Best Regards**

## References

[1] Alagöz C, Yıldırım Y, Kocamaz M, Baz Ö, Çiçek U, Çelik B, Demirkale Hİ, Yazıcı AT, Taşkapılı M (2016). The Efficacy of Intravitreal Bevacizumab in Vitreous Hemorrhage of Diabetic Subjects. Turk J Ophthalmol.

[2] Gross JG, Glassman AR, Jampol LM, Inusah S, Aiello LP, Antoszyk AN, Baker CW, Berger BB, Bressler NM, Browning D, Elman MJ, Ferris FL, Friedman SM, Marcus DM, Melia M, Stockdale CR, Sun JK, Beck RW;, Writing Committee for the Diabetic Retinopathy Clinical Research Network (2015). Panretinal Photocoagulation vs Intravitreous Ranibizumab for Proliferative Diabetic Retinopathy: A Randomized Clinical Trial. JAMA.

[3] Zehetner C, Kirchmair R, Huber S, Kralinger MT, Kieselbach GF (2013). Plasma levels of vascular endothelial growth factor before and after intravitreal injection of bevacizumab, ranibizumab and pegaptanib in patients with agerelated macular degeneration, and in patients with diabetic macular oedema. Br J Ophthalmol.

[4] Virgili G, Parravano M, Menchini F, Brunetti M (2012). Antiangiogenic therapy with anti-vascular endothelial growth factor modalities for diabetic macular oedema. Cochrane Database Syst Rev.
